# Endothelin B Receptors Contribute to Retinal Ganglion Cell Loss in a Rat Model of Glaucoma

**DOI:** 10.1371/journal.pone.0043199

**Published:** 2012-08-20

**Authors:** Alena Z. Minton, Nitasha R. Phatak, Dorota L. Stankowska, Shaoqing He, Hai-Ying Ma, Brett H. Mueller, Ming Jiang, Robert Luedtke, Shaohua Yang, Colby Brownlee, Raghu R. Krishnamoorthy

**Affiliations:** 1 Department of Cell Biology and Anatomy, University of North Texas Health Science Center, Fort Worth, Texas, United States of America; 2 Department of Pharmacology and Neuroscience, University of North Texas Health Science Center, Fort Worth, Texas, United States of America; Dalhousie University, Canada

## Abstract

Glaucoma is an optic neuropathy, commonly associated with elevated intraocular pressure (IOP) characterized by optic nerve degeneration, cupping of the optic disc, and loss of retinal ganglion cells which could lead to loss of vision. Endothelin-1 (ET-1) is a 21-amino acid vasoactive peptide that plays a key role in the pathogenesis of glaucoma; however, the receptors mediating these effects have not been defined. In the current study, endothelin B (ET_B_) receptor expression was assessed in vivo, in the Morrison's ocular hypertension model of glaucoma in rats. Elevation of IOP in Brown Norway rats produced increased expression of ET_B_ receptors in the retina, mainly in retinal ganglion cells (RGCs), nerve fiber layer (NFL), and also in the inner plexiform layer (IPL) and inner nuclear layer (INL). To determine the role of ET_B_ receptors in neurodegeneration, Wistar-Kyoto wild type (WT) and ET_B_ receptor-deficient (KO) rats were subjected to retrograde labeling with Fluoro-Gold (FG), following which IOP was elevated in one eye while the contralateral eye served as control. IOP elevation for 4 weeks in WT rats caused an appreciable loss of RGCs, which was significantly attenuated in KO rats. In addition, degenerative changes in the optic nerve were greatly reduced in KO rats compared to those in WT rats. Taken together, elevated intraocular pressure mediated increase in ET_B_ receptor expression and its activation may contribute to a decrease in RGC survival as seen in glaucoma. These findings raise the possibility of using endothelin receptor antagonists as neuroprotective agents for the treatment of glaucoma.

## Introduction

Glaucoma is an optic neuropathy with a world-wide incidence of nearly 60.5 million patients, characterized by optic nerve degeneration, apoptosis of retinal ganglion cells (RGCs), and corresponding visual field defects, which could lead to blindness [1]–[3]. Glaucoma and other neurodegenerative diseases have several points of similarities, such as axonal degeneration, selective loss of neuron populations (RGCs selectively undergo apoptosis) [4]–[8], and glial activation [9]. Elevated intraocular pressure (IOP) is a major risk factor in primary open-angle glaucoma (POAG), which accounts for the majority of glaucoma patients. Apart from its well-known IOP related effects, glaucoma is recognized as a heterogeneous group of multifactorial neurodegenerative diseases with varying etiologies and clinical presentations. Hence, multiple hypotheses have been proposed to explain the pathophysiology of glaucoma, including mechanical stress of elevated IOP, disruption of retrograde transport of neurotrophins [6], ocular ischemia [10]–[12] glutamate-induced excitotoxicity [13], and oxidative stress [14]–[16]. Currently, the mainstay of glaucoma treatment is a IOP-lowering drug. However, reduction of IOP can only slow RGC loss and optic nerve damage, but cannot completely prevent further degeneration [17], [18]. Hence, understanding molecular mechanisms contributing to RGC death can lead to the development of more effective treatments for glaucoma patients [19].

Corroborative evidence from several laboratories suggests that endothelin-1 (ET-1), a vasoactive peptide, has neurodegenerative effects in glaucoma [20]–[22]. However, the exact mechanisms underlying ET-1's actions remain to be elucidated. Studies have shown that ET-1 concentrations are significantly increased in the aqueous humor (AH) of patients with POAG and in animal models of glaucoma [23]–[25]. Both peribulbar and intravitreal administration of ET-1 has been found to produce axon loss and RGC death [26]–[30].

ET-1 exerts its functions via binding to two classes of G-protein coupled receptors, namely endothelin A (ET_A_) and endothelin B (ET_B_) receptors, both of which are abundantly expressed in various ocular tissues [21], [31], [32]. In animal models of glaucoma, studies have shown that there is an increase in ET_B_ receptor mRNA expression in rat retinas as early as 1 day following IOP elevation and persisted up to 8 weeks of ocular hypertension [33]. Another study [34] demonstrated an increased frequency of ET_B_ receptor immunolocalization in human glaucomatous optic nerves, compared to those of age-matched controls. Previous work from our laboratory suggests that the ET_B_ receptor could be a key mediator of ET-1's neurodegenerative effects following intravitreal administration of ET-1 [30]. The purpose of this study was to analyze ET_B_ receptor expression in the retinas of rats with elevated IOP and to determine if RGC loss is attenuated in ET_B_ receptor-deficient transgenic rats.

## Results

### Elevation of intraocular pressure produced an upregulation of ET_B_ receptors in rat retinas

**Figure 1:**
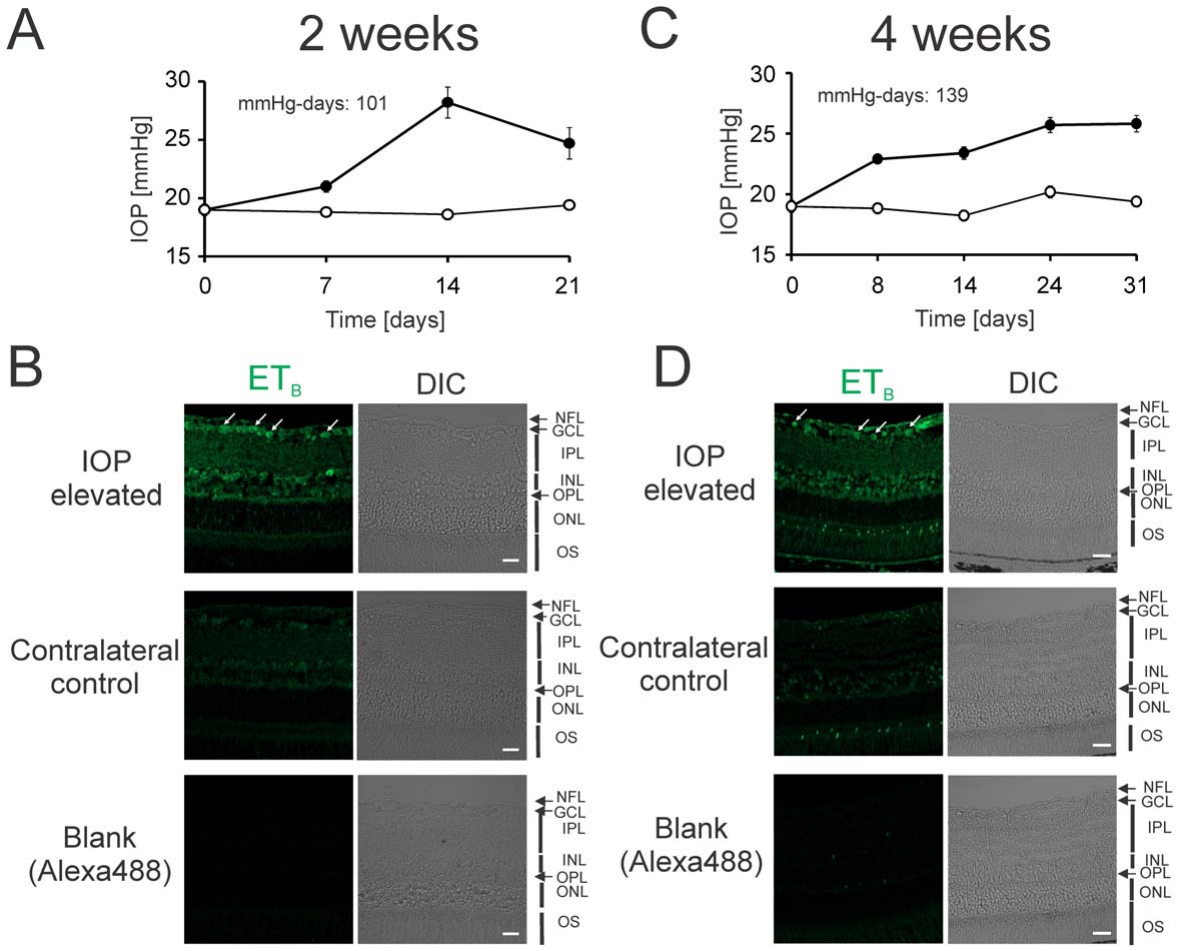
IOP elevation produced a marked increase in ET_B_ receptor expression in rat retinas. Representative IOP elevation profile for 2 weeks (**A**) and 4 weeks (**C**) in adult Brown Norway rats. IOP was elevated in one eye (closed circles), while the other eye served as a contralateral control eye (open circles). The experiment was carried out in six Brown Norway rats for 2 weeks and seven Brown Norway rats for 4 weeks of IOP elevation. IOP values were plotted as mean ± SEM. Retinal sections from 2 weeks (**B**) and 4 weeks (**D**) IOP elevated rat eyes were immunostained for ET_B_ receptor expression (green fluorescence) using a custom made rabbit polyclonal ET_B_ receptor specific antibody. White arrows indicate RGCs in which an increase in immunostaining for ET_B_ receptors was observed. Retinal sections in which the primary antibody incubation was omitted are labeled as blank and showed minimal fluorescence. NFL, nerve fiber layer; GCL, ganglion cell layer; IPL, inner plexiform layer; OPL, outer plexiform layer; INL, Inner Nuclear Layer; OPL, Outer Plexiform Layer; OS, Rod Outer Segment. Scale bar indicates 20 µm.

Previous studies from our laboratory suggested the involvement of ET_B_ receptors in several cellular pathways contributing to neurodegeneration of RGCs [20], [25], [30]. In the present study, we sought to determine whether there are any changes in the ET_B_ receptor expression in rat retinas following IOP elevation for 2 and 4 weeks. Briefly, Brown Norway rats were used to elevate IOP in one eye while the corresponding contralateral eye served as control. Rats were sacrificed after 2 and 4 weeks of IOP elevation and retina sections were obtained from rat eyes. Immunohistochemical analysis of retinal sections from adult Brown Norway rats showed an increased immunostaining for ET_B_ receptors primarily in the nerve fiber layer (NFL) and ganglion cell layer (GCL) in retinas of rats with IOP elevation for 2 weeks (white arrows, Figure 1B), compared to those of the contralateral control eyes. Increased immunostaining for ET_B_ receptors was also observed in inner plexiform layer (IPL), and outer plexiform layer (OPL) in retinas of rats with IOP elevation for 2 weeks, compared to those of the contralateral control eyes (Fig. 1B). A modest increase in immunostaining for ET_B_ receptors was also seen in the inner nuclear layer (INL) after 2 weeks of IOP elevation. Four weeks of IOP elevation also resulted in an increase in ET_B_ receptor expression primarily in the NFL, GCL, IPL, and OPL in retinas of rats with IOP elevation, as compared to control (Fig. 1D). The increase in ET_B_ receptor expression was similar to that seen after 2 weeks of IOP elevation (Fig. 1B). Based upon values of fluorescence intensities (using the Image J software) obtained from confocal images of retinal sections, there a 2.59 fold increase in ET_B_ receptor immunostaining after 2 weeks of IOP elevation and a 2.52 fold increase after 4 weeks of IOP elevation. Interestingly, robust staining for the ET_B_ receptor was also observed in the INL following 4 weeks of IOP elevation.

**Figure 2:**
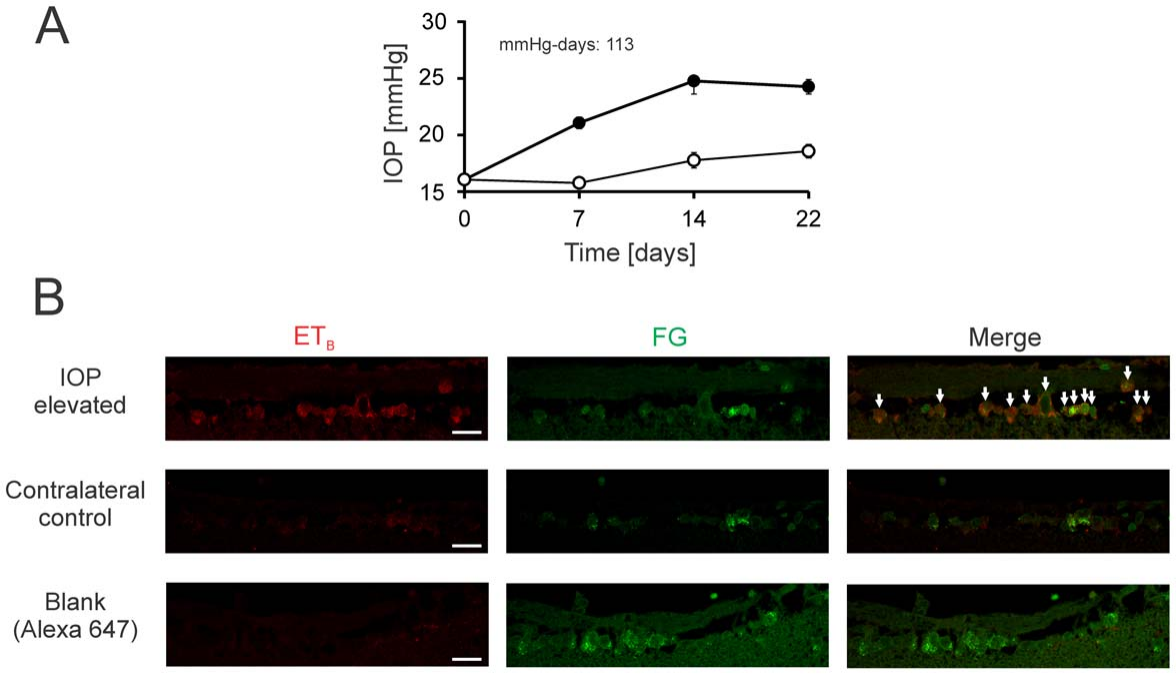
Increased ET_B_ receptor immunostaining in Fluoro-Gold labeled retinal ganglion cells following IOP elevation in rats. **A.** Representative IOP elevation profile for 2 weeks in adult Brown Norway rats. Adult male Brown Norway rats (n = 3) were subjected to retrograde labeling using Fluoro-Gold (FG) to fluorescently label RGCs. IOP elevation was carried out in one eye in these rats and maintained for 2 weeks, while the companion eye served as a contralateral control. **B.** Immunohistochemical staining with a ET_B_ receptor antibody was done using retinal sections from IOP elevated eyes and their corresponding contralateral control eyes and detected with an Alexa 647 conjugated secondary antibody. Fluorescence images were taken in a confocal microscope and merged images of the ET_B_ immunofluorescence with FG was obtained (Merge). Scale bar indicates 20 µm.

To ascertain that an increase in ET_B_ receptor expression occurred in RGCs after IOP elevation, an immunostaining for ET_B_ receptors was performed on retinal sections from Fluoro-Gold (FG) labeled rats. Briefly, retinal ganglion cells of adult male Brown Norway rats were retrogradely labeled with FG, following which IOP was elevated in one eye [35]. After maintaining the rats for 2 weeks following IOP elevation, the animals were sacrificed, retina sections were obtained and immunostained for ET_B_ expression. An increased immunostaining for ET_B_ receptor was observed mainly in retrogradely labeled RGCs in the RGC layer in IOP elevated eyes, compared to the corresponding control eyes (Figure 2B). This provides further confirmation for increased ET_B_ receptor expression in RGCs following IOP elevation in rats.

### ET_B_ receptor binding activity was increased in retinas of rats with elevated IOP

**Figure 3:**
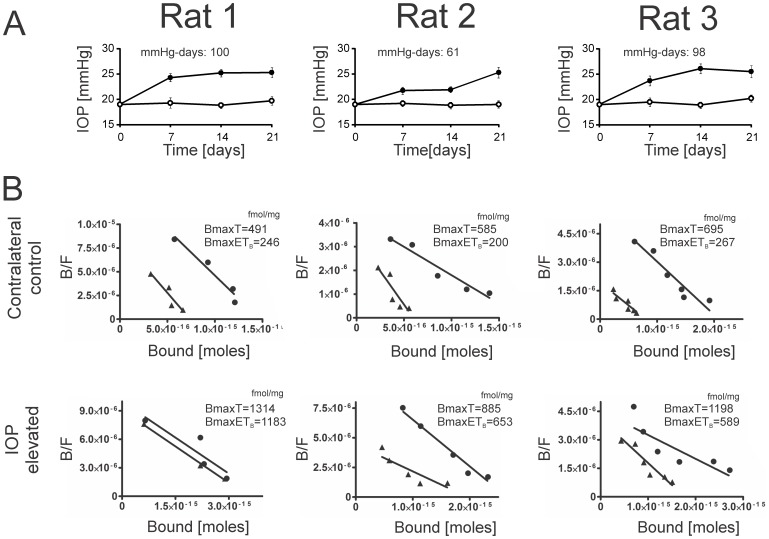
ET_B_ receptor binding activity was increased in retinas of IOP elevated Brown Norway rats. **A.** IOP elevation profile in adult male Brown Norway rats. IOP was elevated in one eye (treated eye: closed circles), while the other eye served as the contralateral control eye (open circles). The experiment was carried out in three Brown Norway rats (n = 3) represented by Rat 1, Rat 2, and Rat 3. **B.** Scatchard plots of binding of ^125^I-ET-1 to rat retinal membranes from contralateral control and IOP elevated eyes. Data points represent the means of triplicate binding reactions (Circles represent total endothelin receptor binding, while triangles represent ET_B_ receptor binding). Three individual binding experiments were performed using contralateral control eyes from three rats (Rat 1, Rat 2, and Rat 3). Bmax (fmol/mg) representing total number of binding sites are indicated in the plots. B/F represents the ratio of bound to free radioactive ET-1 ligand. Binding assays for both control and elevated IOP eyes were carried out concurrently using the same conditions.

Since an increase in ET_B_ proteins was observed by immunostaining in IOP elevated rat retinas, further confirmation was made by receptor binding assays using radiolabeled ET-1. In preliminary experiments ^125^I-labeled ET-1 was found to bind to rat retinal membrane (3 µg of protein) in a linear pattern. The ET_A_ receptor antagonist, BQ-610, yielded a Ki value of 16.3 nM in competitive binding assays using rat retinal membranes. This experimental Ki is very close to the Ki value (20 nM) provided for BQ-610 by the manufacturer (Peninsula Lab Inc. Belmont, CA, USA). Based on the Ki value, BQ-610 was used at a concentration of 200 nM in the receptor binding assays Three individual binding assays were performed to plot endothelin receptor binding sites in the presence of 0.2–2 nM of ^125^I-ET-1, 1 µM of unlabeled ET-1 and 200 nM of BQ-610. The number of binding sites, Bmax (fmol/mg), of total endothelin receptors (ET_A_ and ET_B_ receptors) and ET_B_ receptors was calculated for retinas from elevated IOP and contralateral eyes using unweighted linear regression analysis after normalization to the amount of protein by the method of Scatchard [36]. Total specific endothelin receptor binding (Bmax values) in retinal membranes from IOP-elevated eyes increased 2.7-, 1.5- and 1.7-fold respectively (from three independent binding assays) compared to the binding in the contralateral eye (Figure 3B). There was a significant (p<0.05) mean 1.9-fold increase in the total specific endothelin receptor binding activity (Bmax values) in the IOP elevated eyes, compared to the contralateral eyes (Table 1). On the other hand, there was no significant change in the binding affinity (reflected by the Kd values) for the total endothelin binding between control and elevated IOP eyes (Table 2). Specific ET_B_ receptor binding (Bmax values) was increased 4.8-, 3.3- and 2.2-fold in the IOP-elevated eye, compared to the corresponding contralateral eye (Figure 3B). There was a significant (p<0.05) mean 3.3-fold increase in specific ET_B_ receptor binding in IOP elevated eyes, compared to control eyes (Table 1). The Kd values for the specific ET_B_ receptor binding were not significantly different between control and IOP elevated eyes (Table 2). The results indicate that number of endothelin receptors was increased in the retina from IOP-elevated eye, and in particular ^125^I-ET-1 binding to ET_B_ receptors was even higher in IOP elevated eyes, compared to contralateral eyes.

**Table 1:** Bmax values (fmol/mg) for endothelin receptor binding.

	Rat No. 1	Rat No. 2	Rat No. 3	Mean ± SD
Control (Total Binding)	491	585	695	590±83.4
Control (ET_B_ receptor binding)	246	200	267	238±28
Elevated IOP (Total Binding)	1314	885	1198	1132±181*
Elevated IOP (ET_B_ receptor binding)	1183	653	589	808±266*

* indicates statistical significance (p<0.05) by Student's t-test.

**Table 2:** Kd values (pM) for endothelin receptor binding.

	Rat No. 1	Rat No. 2	Rat No. 3	Mean ± SD
Control (Total Binding)	103	419	358	293±136.87
Control (ET_B_ receptor binding)	86	177	414	225±138.26
Elevated IOP (Total Binding)	395	253	798	482±230.8
Elevated IOP (ET_B_ receptor binding)	390	443	432	421±22.8

### Retinal ganglion cell loss was attenuated in ET_B_ receptor-deficient transgenic rats after 4 weeks of IOP elevation

**Figure 4:**
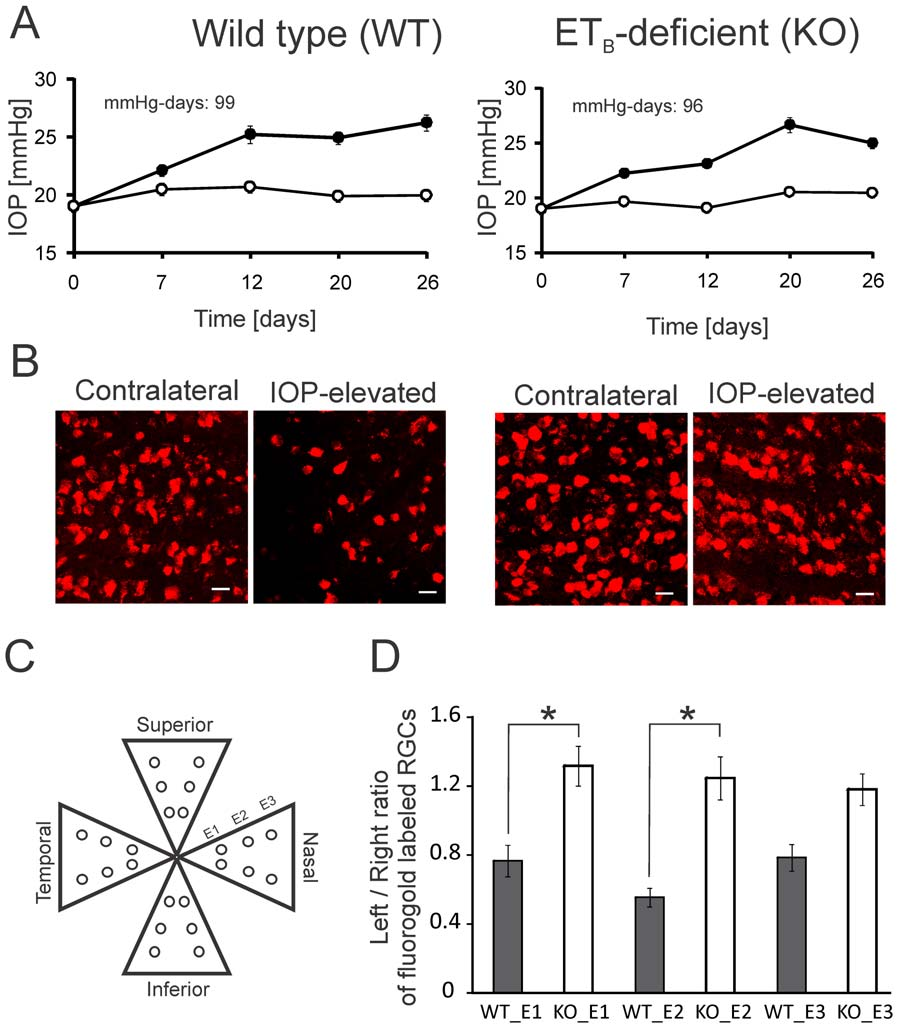
RGC loss following IOP elevation was attenuated in KO rats compared to WT rats. **A.** IOP elevation profile in WT and KO rats. Following FG labeling, IOP was elevated in one eye (closed circles), while the other eye served as the contralateral control eye (open circles). IOP values are plotted as mean ± SEM. **B.** Fluorescent images of retrograde labeled retinal ganglion cells in contralateral and IOP elevated retinas from WT and KO rats. **C.** Scheme for analysis of RGC counts in three eccentricities (E1, E2 and E3) in four quadrants of retinal flat mounts. **D.** Plot of ratio of Fluoro-gold labeled RGCs between left (IOP elevated) and right (contralateral) eyes in different eccentricities. The ratio was compared between WT and KO rats for three eccentricities (E1, E2 and E3). A significant increase in RGC survival was observed in KO rats (n = 4) as compared to WT rats (n = 3). Bars represent mean ± SEM. * indicates significance (p<0.05) by ANOVA on ranks followed by pair-wise multiple comparisons (Dunn's method). Scale bar indicates 20 µm.

IOP elevation in the Morrison rat model of glaucoma has been shown to result in a significant increase in TUNEL-positive RGCs, axonal loss, and gliosis [37]–[39]. However, the key contributors to RGC death are not completely understood. The endothelin family of peptides has recently gained prominence for their neurodegenerative effects in the retina [28], [30], [40]; however, the receptors mediating these effects have not been identified. To determine the involvement of ET_B_ receptors in neurodegenerative effects, RGCs of wild type (WT) and ET_B_ receptor-deficient transgenic rats (KO) were retrogradely labeled with FG, following which IOP elevation was carried out and maintained for 4 weeks (Figure 4A). After sacrificing the animals, retinal flat mounts were obtained and RGC survival was assessed by counting viable RGCs (Figure 4B) in three eccentricities (E1, E2 and E3) within each retinal quadrant (Figure 4C). The loss of RGCs due to IOP elevation was computed by calculating the ratio of RGC counts between left (IOP elevated) and right (control) eye for each eccentricity. The ratio of RGC counts between left and right eye for each eccentricity was then compared between WT and KO rats (Figure 4D). Interestingly, the ratios of RGC counts between left and right eye were significantly higher in KO rats in the first two eccentricities (E1 and E2), compared to those of the WT rats (Figure 4D). The ratio of RGCs counts between left and right eyes was also higher in the third eccentricity of the KO rats; however, it did not attain statistical significance (Figure 4D). These results demonstrate that the ET_B_ receptor may play a causative role in RGC death following elevation of IOP, and blocking this receptor may aid in neuroprotection of RGCs.

### Axonal integrity was maintained in ET_B_-deficient rats after IOP elevation

**Figure 5:**
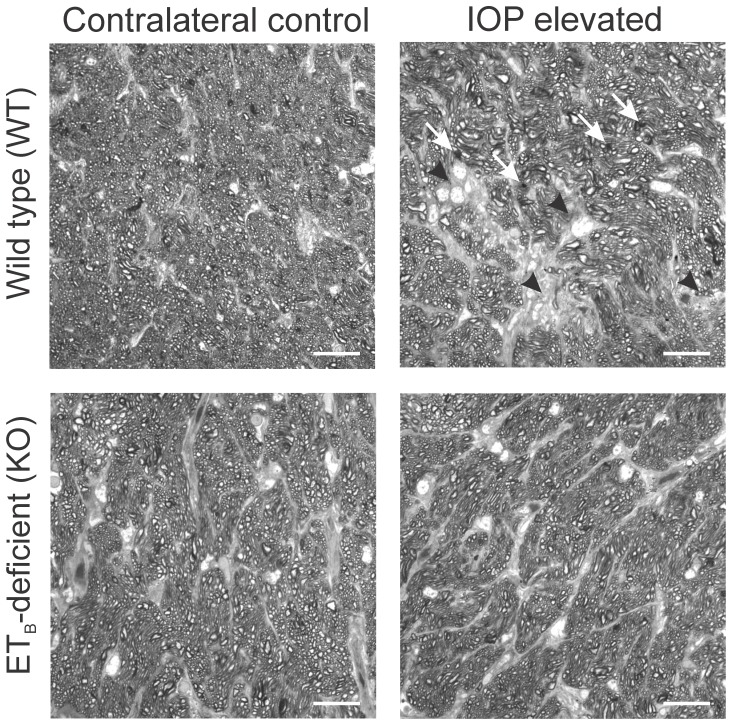
Survival of optic nerve axons in KO rats, compared to WT rats following IOP elevation. IOP was elevated in one eye of WT and KO rats, while the other eye served as corresponding contralateral control. The rats were maintained for 4 weeks following IOP elevation. Rats were sacrificed and optic nerve sections were stained with paraphenylenediamine. Dark spots (white arrows) indicate dying/degenerating axons. Glial scar formation (black arrow heads) was more abundant in WT rats, compared to KO rats in IOP elevated eyes. Scale bar indicates 20 µm.

Since a significant protection of RGCs was observed in KO rats subjected to IOP elevation, compared to WT rats, optic nerve axonal integrity was assessed in these animals. IOP was elevated in one eye of WT and KO rats, while the corresponding contralateral eye served as control [35]. After IOP elevation, rats were maintained for 4 weeks, sacrificed and optic nerve sections were obtained and stained with paraphenylenediamine (PPD). As seen in Figure 5, an increased staining with PPD was observed in optic nerve sections from IOP elevated WT rats. In addition, disruption of axon bundles, increased gliosis and glial scar formation was seen in the optic nerve sections from WT rats following IOP elevation. In contrast, optic nerve sections from IOP elevated KO rats showed a better preservation of axon morphology (Figure 5). Glial scarring was less prominent in KO rats compared to that seen in WT rats following IOP elevation. Optic nerve grading was done by masked observers essentially according to the method described by Chauhan et al. (2006). Grade 0 was assigned to optic nerves with no damage with all the nerve bundles intact, while grades 3 and 6 correspond to nearly 30% and 60% of mean damage. The analysis indicated that there was no appreciable difference between the integrity of optic nerve between contralateral control eyes of the WT and KO rats (graded 1 to 2). However, the optic nerve sections from IOP elevated KO rat eyes showed lesser damage (graded 2 to 3) compared to optic nerves from the IOP elevated WT rats (graded 3 to 4). These observations suggest that lack of ET_B_ receptor activation could have protective effects on the axons of the optic nerve in addition to preventing RGC loss.

### ET-1 treatment produced increased cell death in cultured primary retinal ganglion cells

**Figure 6:**
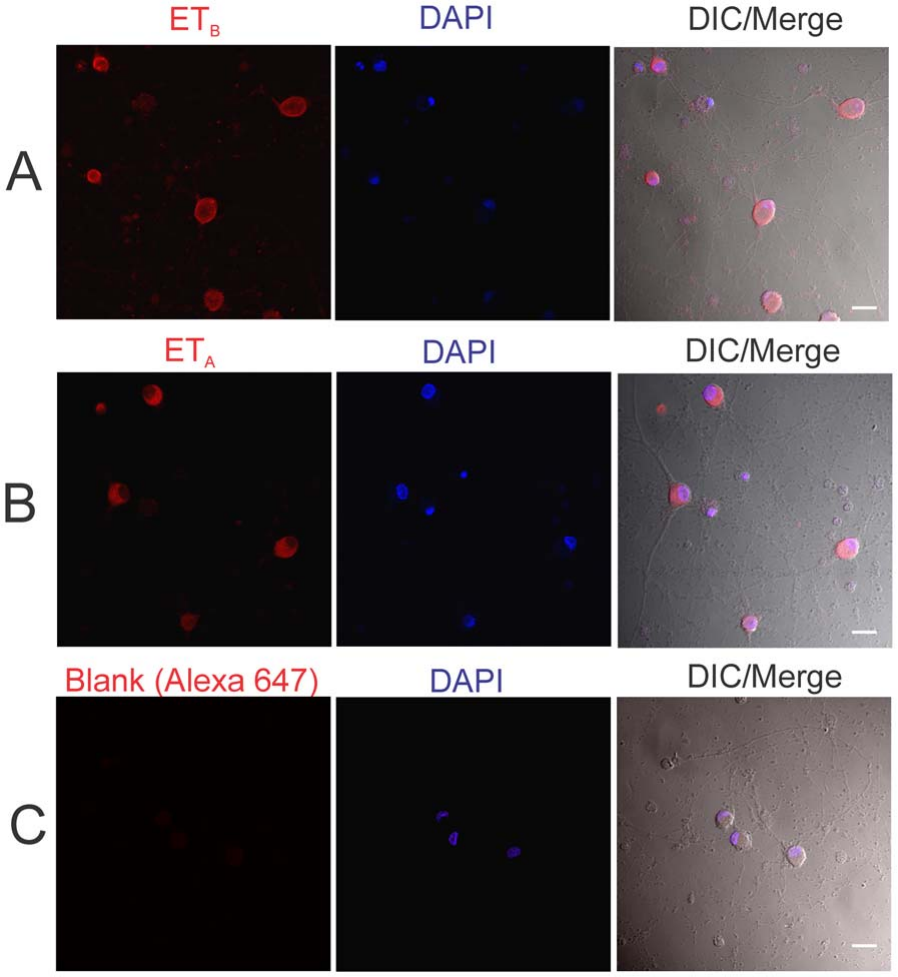
Immunocytochemical analysis of endothelin receptor expression in primary rat retinal ganglion cells (RGCs). Primary rat RGCs were isolated from post-natal day 3–7 rat pups and immunocytochemical analysis of (**A**) ET_B_ and (**B**) ET_A_ expression was performed using a custom-made ET_B_ antibody and a commercially available ET_A_ antibody. The immunostaining was detected using corresponding Alexa 647 conjugated secondary antibodies. (**C**) A negative control immunostaining (Blank) in which the primary antibody was omitted showed minimal staining. Cells were counterstained with DAPI to detect cell nuclei. Scale bar indicates 20 µm.

**Figure 7:**
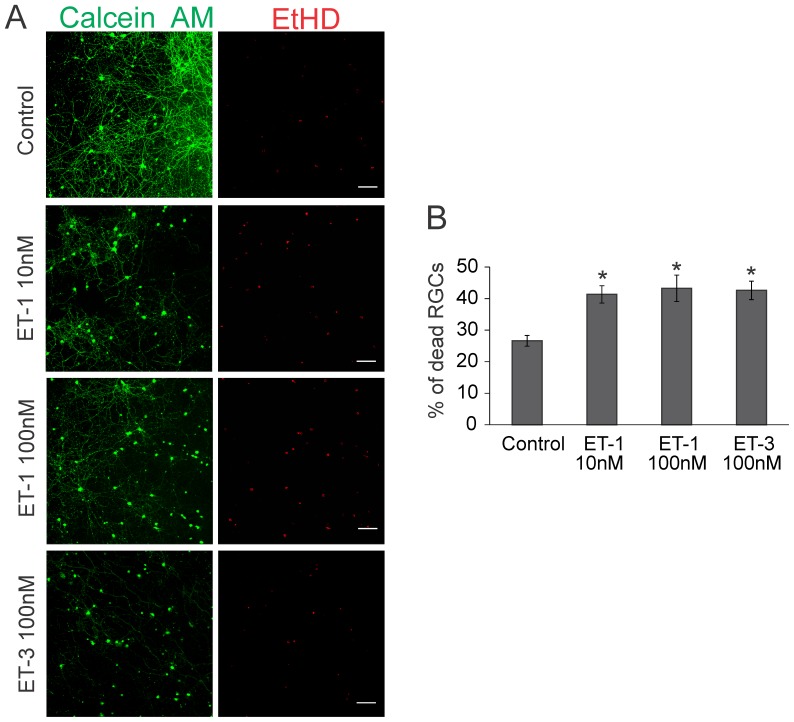
Live-dead assay of primary RGCs treated with endothelins. **A.** Primary retinal ganglion cells were obtained from post-natal day 3–7 rat pups and seeded on coverslips. The cells were either untreated (control) or treated with 10 nM ET-1, 100 nM ET-1 or 100 nM ET-3 for 24 hrs. Following the treatments, a mixture of calcein AM (green fluorescence indicating living cells) and Ethidium homodimer (EtHD) (red fluorescence indicating dead cells) was added and cell viability assessed. **B.** Plot of percentage of dead cells after treatment of primary RGCs with ET-1 (10 nM), ET-1 (100 nM) and ET-3 (100 nM). The plot represents the average of three independent experiments and bars represent mean ± SEM. * indicates statistical significance (p<0.05) by Kruskal-Wallis One Way ANOVA on Ranks followed by Dunn's multiple comparison. Scale bar indicates 100 µm.

Primary cultures of RGCs were isolated and were first tested by immunocytochemistry to determine if they express ET_B_ and ET_A_ receptors. Immunostaining for ET_B_ and ET_A_ receptors was observed both in the soma and neurites of cultured primary RGCs (Figure 6A, B). Immunocytochemical analysis of primary RGCs in which the primary antibody was excluded (Blank) showed minimal staining indicating that there was no appreciable non-specific binding of the Alexa647 conjugated secondary antibody (Figure 6C). To determine if endothelin receptor activation could promote death of RGCs, primary RGCs grown on coverslips were treated with two different concentrations of ET-1 (10 nM and 100 nM) or with the ET_B_ receptor agonist, ET-3 (100 nM). Another group of RGCs grown on coverslips that did not undergo any treatment served as controls. Following the treatments, a mixture of green-fluorescent calcein-AM (indicative of intracellular esterase activity of viable cells) and red-fluorescent ethidium homodimer-1 (EtHD) (indicative of loss of plasma membrane integrity of dead cells) was added to assess the viability and death of the cells respectively. As seen in figure 7A, untreated RGCs had good morphology as evidenced by multiple neurites from each cell and forming a network of synaptic connections with neighboring cells. The cells were brightly stained with calcein-AM (green fluorescence) indicating that they were viable. There were also some EtHD stained cells (Mean ± SEM: 27±1.7% of cells) indicative of cell death in the untreated cells, possibly due to loss of trophic support in these cells. In cells treated with 10 nM ET-1 there was a decrease in staining with calcein AM and a withdrawal of neurites from many cells. The number of EtHD stained cells, indicative of cell death significantly increased in the 10 nM ET-1 treated cells (Mean ± SEM: 41±2.7% of cells), compared to the controls. The cell viability was further exacerbated in primary RGCs when treated with 100 nM ET-1. These cells exhibited fewer processes, compared to the 10 nM treatment, and nuclei were found to be more condensed indicative of apoptotic changes (Figure 7A). Moreover, RGCs treated with 100 nM ET-1 showed significantly higher EtHD staining (Mean ± SEM: 43±4.2% of cells), compared to untreated control cells (Figure 7B). Primary RGCs treated with the ET_B_ receptor agonist, ET-3, demonstrated almost complete withdrawal of neurites and also showed significantly higher number of EtHD staining indicative of cell death, compared to controls (Mean ± SEM: 43±2.9% of cells) (Figure 7B). These results suggest that ET-1 acting predominantly through the ET_B_ receptor promotes cell death of RGCs.

### ET-1 treatment promoted cell death via apoptosis in primary retinal ganglion cells

**Figure 8:**
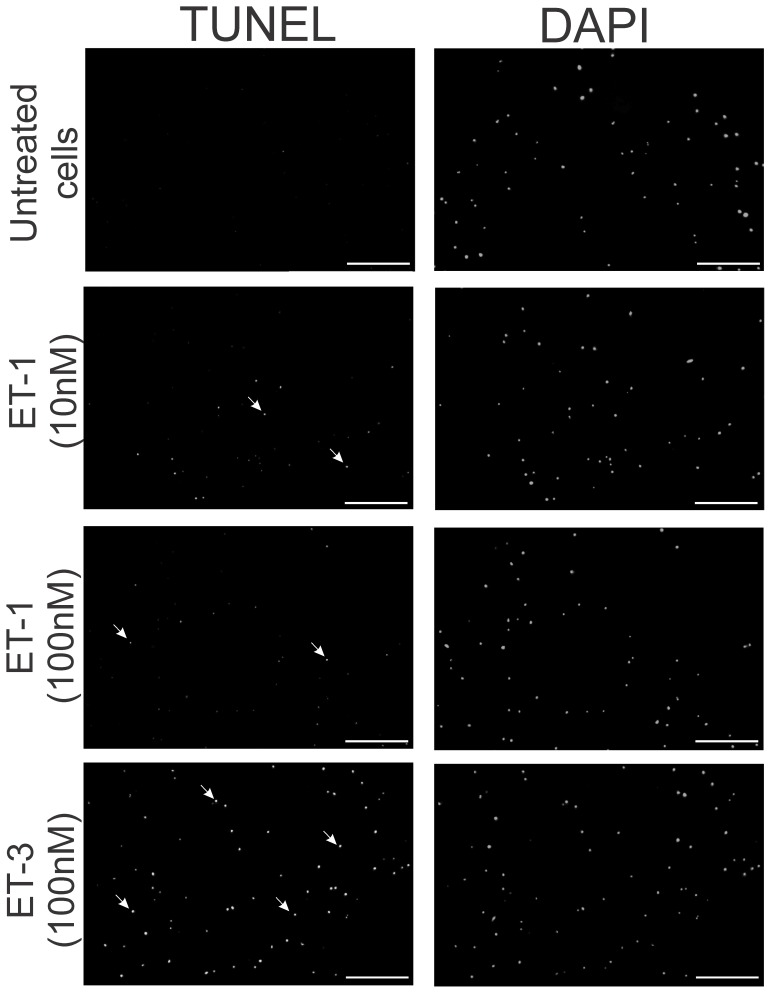
TUNEL assay of primary RGCs treated with endothelins. Primary RGCs were either untreated or treated with ET-1 (10 nM), ET-1 (100 nM) or ET-3 (100 nM) and TUNEL assays were carried out to detect apoptosis. The left vertical panel (TUNEL) indicates fluorescent images from cells incorporating fluorescinated dUTP indicative of apoptosis. The right vertical panel (DAPI) shows stained nuclei using DAPI. Scale bar indicates 200 µm.

Since ET-1 treatment produced increased cell death of primary retinal ganglion cells, further experiments were carried out to ascertain if this occurred through an apoptotic mechanism. To test this, primary RGCs were isolated from post natal day 3–7 rat pups, seeded on 12 mm coverslips and allowed to attach and grow for 1 week. The RGCs were either untreated or treated with ET-1 (10 nM), ET-1 (100 nM) or ET-3 (100 nM) for 24 hr. After the treatments, cells were fixed with 4% paraformaldehyde for 25 min at 4°C and TUNEL assays were carried out to detect apoptotic cell death using a commercially available kit from Promega (Madison, WI, USA) as per the manufacturer's instructions. As seen in Figure S1, RGCs treated with a reaction mix in which the TdT enzyme was excluded (negative control) showed no staining whereas cells treated with DNAse I to introduce artificial strand breaks (positive control) yielded a robust TUNEL positive reaction, ensuring the validity of the TUNEL assay. Untreated RGCs showed minimal staining for TUNEL positive cells, indicating that there was no appreciable apoptotic cell death in the untreated RGCs (Figure 8). However, in both 10 nM and 100 nM ET-1 treatment increased TUNEL labeling was observed, indicative of apoptosis (Figure 8). The most intense TUNEL labeling was observed in primary RGCs treated with the ET_B_ receptor agonist, ET-3, suggesting that the ET_B_ receptor could be a key mediator of apoptosis in RGCs (Figure 8).

## Discussion

An accumulating body of evidence suggests the involvement of ET-1 [20], [21], [41]–[43] and ET_B_ receptors [25], [30], [33], [34], [42], [44]–[46] in the pathogenesis of glaucoma, however a causal link of ET_B_ receptors to neurodegeneration has not been clearly established.

The endothelin family is comprised of three endothelin isoforms, namely ET-1, ET-2, and ET-3, each of which is encoded by different genes [47], [48]. ET-1 is the most studied isoform, which is constitutively synthesized and secreted from several tissues. Originally purified and characterized from vascular endothelial cells [49], ET-1 has been shown to be present in several tissues and organs including the eye [50]–[52] and brain [53], [54]. The normal physiological role of ET-1 in the central nervous system remains to be understood. However, studies have demonstrated that ET-1 is a key player in various neurodegenerative conditions, including Alzheimer's disease, retinal degeneration and glaucoma [22], [40], [55], [56]. In Parkinson's disease, the Parkin-associated endothelin receptor-like receptor (Pael-R) has been shown to induce unfolded protein response (UPR)-mediated cell death [57]. Nevertheless, the detailed mechanisms underlying ET-1's neurodegenerative effects remain to be elucidated.

ET-1 exerts its functions via binding to two classes of G-protein coupled receptors, ET_A_ and ET_B_ [21], [31], [32]. The two receptors have different affinities for the different endothelin peptides. The ET_A_ receptor has equal affinity for ET-1 and ET-2 peptides, but much lower affinity for ET-3 (ET_A_: ET-1 = ET-2 » ET-3), while the ET_B_ receptor has equal affinity for all the three endothelin peptides (ET_B_: ET-1 = ET-2 = ET-3). Thus, ET-3 could be used as an ET_B_ receptor agonist. Clinical studies have found increased levels of ET-1 in aqueous humor of primary open angle glaucoma patients [58] and increased circulating levels of ET-1 in normal tension glaucoma patients [59]. ET-1 concentrations were also found to be elevated in exfoliation syndrome (the production and progressive accumulation of a fibrillar extracellular material in many ocular tissues) [60], [61]. ET_A_-like receptor binding sites have been identified in the retina and choroidal blood vessels [31]. In contrast, ET_B_-like receptor-binding sites were primarily found in the neural and glial components of the retina [32], [62]. Studies have demonstrated that primary cultures of human trabecular meshwork, ciliary muscle, and ciliary nonpigmented epithelial cells predominately express the ET_A_ receptor [62].

Since ET_B_ receptor expression increases at very early time points of IOP elevation (when no appreciable loss of RGCs occurs) [33], it could play a causative role in neurodegeneration in glaucoma. Interestingly, we found a profound increase in ET_B_ receptor expression in the nerve fiber layer (NFL) and ganglion cell layer (GCL) in retinas of rats with IOP elevation for 2 weeks, compared to those of the contralateral control eye (Fig. 1B) and this elevation in ET_B_ receptor expression was maintained up to 4 weeks of IOP elevation. Since RGCs are located in the GCL along with displaced amacrine cells [63]–[65], it is important to distinguish between these two cell populations to conclude that changes in ET_B_ receptor expression occur primarily in RGCs in IOP elevated eyes. An increased staining of ET_B_ receptors was observed mainly in the FG labeled RGCs in the RGC layer after IOP elevation for 2 weeks (Figure 2B). We also observed an increase in the expression of ET_B_ receptors in the inner plexiform layer (IPL) and outer plexiform layer (OPL) in retinas of rats with IOP elevation for 2 weeks, in comparison to contralateral control eye (Fig. 1B). It is known that IPL is the layer where the bipolar cell axons form synapses with dendrites of the ganglion and amacrine cells, whereas OPL contains projections of rods and cones [66]. Interestingly, increased staining for ET_B_ receptors was observed in the inner nuclear layer (INL) following 4 weeks of IOP elevation INL, which is composed the cell bodies of the bipolar, horizontal, and amacrine cells (Figure 1D). The significance of upregulation of ET_B_ receptors in these retinal layers is currently unclear, however these cell types do not undergo pathological changes in glaucoma.

Receptor-binding assays using ^125^I-ET-1 showed a preferential upregulation of ET_B_ receptors in the retina following IOP elevation. A significant increase in the Bmax values (indicative of number of receptor binding sites) for ET_B_ receptor binding was observed without a significant increase in the corresponding Kd values (indicative of receptor affinity), which is a classic pharmacological measure of upregulation of receptor expression. ET_B_ receptors function as clearance receptors and play a critical role in maintaining normal levels of ET-1 [67], [68]. It is possible that expression of ET_B_ receptors is increased in response to elevation of ET-1 concentrations following IOP elevation. Increased expression of ET_B_ receptors was also detected at the level of the optic nerve head in animal models, as well as in glaucoma patients [25], [30], [34]. In animal models of photoreceptor degeneration, another member of the endothelin family, ET-2, was found to be elevated and accompanied by more than 10-fold increase in ET_B_ expression primarily in the glial cells in the retina [40].

In glaucoma, RGCs are selectively lost via apoptosis [7], [69]–[73], while other retinal neurons are largely unaffected. However, the key contributors to RGC death have not been completely identified. To assess RGC viability in vivo, we used retrograde labeling with FG, which has been shown to label 98.4% and 97.8% of the RGCs in albino and pigmented rats, respectively [74]. Using this method followed by IOP elevation, it was found that four weeks of IOP elevation in WT rats produced a significant loss of RGCs. In contrast, loss of RGCs was significantly attenuated (p<0.05) in the first two eccentricities in retinas of KO rats, compared to those of WT rats. Apart from RGCs, neuroprotective effects were also observed in the axons of KO rats in comparison to WT rats following IOP elevation. Considering the robust neuroprotection observed in KO rats, it is possible that ET_B_ receptor activation in multiple cell types (including lamina cribrosa, RGCs and optic nerve head astrocytes) may be contributing to neurodegeneration in glaucoma. Thus, blocking ET_B_ receptors could have additive neuroprotective effects due to the involvement of ET_B_ receptors in pathological changes in several tissues in glaucoma.

Using primary RGCs, it was found that ET-1 treatment at two different concentrations (10 and 100 nM) produced a significant increase in cell death, suggesting that ET-1 acting through its receptors produces neurodegenerative effects. Since ET-3, an ET_B_ receptor agonist, produced a similar extent of cell death as seen with ET-1 (which acts on both ET_A_ and ET_B_ receptors), it is possible most of endothelin-1 mediated degenerative effects on RGCs occur through the ET_B_ receptors. Using the TUNEL assay, it was found that the mode of cell death by ET-1 treatment was via apoptosis in primary RGCs. The ET_B_ receptors appeared to play a key role in apoptotic cell death of RGCs since the most intense TUNEL labeling was observed in primary RGCs treated with an ET_B_ receptor agonist (ET-3) (Figure 8). In a recent study, bosentan treatment (which blocks both ET_A_ and ET_B_ receptors) was shown to protect against axonal degeneration in the DBA/2J mouse model of glaucoma, suggesting that some of the degenerative effects could also occur through the ET_A_ receptor [75].

In conclusion, we have found that upregulation of ET_B_ receptors in RGCs occurs at early stages in the Morrison's ocular hypertension rodent model of glaucoma, and may contribute to the death of RGCs. Endothelin receptor antagonists could be promising candidates for neuroprotection in glaucoma.

## Materials and Methods

### Animals

All animal experiments were performed in accordance with the Association for Research in Vision and Ophthalmology (ARVO) policy on the Use of Animals in Vision Research, and all protocols were reviewed and approved by the institutional animal care and use committee (IACUC) at the University of North Texas Health Science Center. Adult male retired breeder Brown Norway (Rattus norvegicus) rats (200–350 g) were purchased from Charles River (Wilmington, MA). Wistar-Kyoto ET_B_ receptor-deficient transgenic rats (KO) (a kind gift from Dr. Masashi Yanagisawa, UT Southwestern Medical Center, Dallas, Texas) were maintained in the vivarium at the UNT Health Science Center. The rats were rescued transgenic ET_B_-deficient spotting lethal (^TG^ET_B_^sl/sl^) rats. The parental heterozygous spotting lethal strain carries a naturally occurring deletion in the first exon of the ET_B_ receptor gene that completely abrogates expression of a functional ET_B_ receptor. Deletion of the ET_B_ receptor in rats is lethal beyond the first few weeks after birth due to aganglionic intestinal obstruction. However, the ET_B_ receptor-deficient transgenic rats used in this study had been rescued by tissue-specific expression of the ET_B_ receptor in the intestine, mediated by the dopamine-β-hydroxylase promoter linked to a functional ET_B_ transgene, which directs ET_B_ expression specifically to the intestine. This allows for normal development of the enteric nervous system; thus, preventing neonatal lethality in these ET_B_-deficient rats [76]. Heterozygous breeding pairs were used to produce offsprings typically with a Mendelian ratio of genotypes. We performed routine genotyping of rat pups to ensure the genotypes of the WT and KO rats that were used in these studies. Rats were maintained under constant low illumination (90 lux).

### Retrograde labeling with Fluoro-Gold (FG)

Retrograde labeling of RGCs was carried out in WT and KO rats as described earlier [77]. The animals were anesthetized with intraperitoneal injection (i.p.) of an anesthesia cocktail comprising of 50 mg/ml ketamine, 5 mg/ml xylazine and 1 mg/ml acepromazine (100 µl/100 g body weight) and placed into a stereotaxic frame (Tujunga, CA, USA) to secure the cranium. Double injections of FG (Denver, CO, USA), were carried out using two sets of stereotaxic coordinates: (i) anterior posterior (AP) = 5.8, ML = +1.3, DV = 3.5 and (ii) AP = 5.8, ML = −1.3, DV = 3.5 from the bregma. For each injection, approximately 3 µl of FG (2% solution in isotonic saline) were injected using a 10 µl Hamilton syringe (Reno, NV, USA) at a rate of 1 µl/min. Following recovery for 2 weeks, IOP was elevated in one eye of each rat by injection of hypertonic saline through episcleral veins [35].

### Morrison's ocular hypertension model of glaucoma in rats

The procedure of Morrison et al. (1997) was used to elevate IOP in rats [35]. Initial studies were carried out in male Brown Norway retired breeder rats to study the effect of elevated IOP on expression of ET_B_ receptors in the retina. To test the role of ET_B_ receptors in neurodegeneration, wild type and ET_B_ receptor-deficient transgenic rats were used after IOP elevation. Animals were maintained on a reduced constant light environment of 90 lux for a minimum of 3 days prior to surgery for elevating IOP. Daily IOP measurements using a Tonolab tonometer (Icare Finland Oy, Espoo, Finland), conducted on the conscious animals after slight sedation with intramuscular (i.m.) administration of acepromazine. On the day of the surgery, animals were anesthetized with an i.p. injection of a standard rat cocktail consisting of ketamine, xylazine, and acepromazine. One eye of each animal was injected with 1.8 M hypertonic saline via an episcleral vein, while the contralateral eye served as a control. A micro glass needle was inserted into the episcleral vein and approximately 50 µl of hypertonic saline was injected with a force sufficient to blanch the aqueous plexus. This procedure produces scarring of the trabecular meshwork with a resultant rise in IOP and damage to the optic nerve [35]. The rats were sacrificed by overdose with pentobarbital (administered first intraperitoneal and then intracardial) at two different time points (2 and 4 weeks) following IOP elevation.

### IOP measurements

IOP was measured in conscious animals using Tonolab tonometer (iCare, Finland). Rats were slightly sedated by an intramuscular injection of acepromazine (2 mg/kg) and IOP measurements were taken 2 to 5 min after the injection. During each IOP measurement session, ten average readings from control and IOP-elevated eyes were obtained. A plot of mean IOP versus time was carried out and IOP exposure was calculated as the number of mmHg days by performing a separate “area-under the curve” (AUC) integration of IOP over the days of exposure for the treated and control eye in each rat [78]. The integral IOP value of the control eye was subtracted from the integral value of the IOP-elevated eye to give the “IOP-integral difference” which was expressed as mmHg days.

### Quantification of RGC survival

Retrograde labeling of retinal ganglion cells using FG was carried out in rats as described by Husak et al. (2000). Two weeks following retrograde labeling, IOP was elevated in the left eye, while the right eye served as the contralateral control eye. After IOP elevation for 4 weeks, rats were sacrificed using intraperitoneal injection of pentobarbital (120 mg/kg), and the orientation of each eye was marked. Eyes were enucleated and immersion-fixed in 4% paraformaldehyde (PFA) (Phillipsburg, NJ, USA) in 0.1 M sodium phosphate, pH 7.2, for 3 h at room temperature. The retinas were dissected and flat-mounted in fluorosave reagent (Calbiochem, USA). Fluorescent images of the retinal flat mounts were taken using a Zeiss LSM 510 META confocal microscope.

FG-labeled cells were manually counted by a blinded observer using fluorescent images obtained from the confocal microscope. Each retina was divided into four quadrants: superior, inferior, nasal, and temporal. Six pictures were taken at 40× magnification in each retinal quadrant. Briefly, the number of RGCs was counted in a blinded manner in 6 areas per retinal quadrant at three different eccentricities (E1, E2 and E3) located at 2/6, 4/6, and 5/6 of the radius of the retina from the optic nerve head respectively (Figure 4C). A total of 24 pictures were taken from each retina. The number of FG-labeled RGC was counted as relatively round somata with dendritic processes from a slide-projected image with 0.37 to 0.53 mm^2^ area of the retina. The spindle-shaped, FG-positive microglia could be easily recognized from these photographs and were not counted. RGC survival was expressed as a ratio of the counts in the elevated IOP eye to that of the contralateral control eye, for the same eccentricity.

### Immunohistochemistry

Retinal sections from Brown Norway rats were subjected to immunohistochemical detection of ET_B_ receptor expression essentially as described by Krishnamoorthy et al. [30]. Briefly, five-micron saggital retinal sections through the optic nerve head were de-paraffinised in xylene (Fisher Scientific, NJ, USA), re-hydrated using a descending series of ethanol washes. Following permeabilization with 0.1% Triton X-100 and blocking with 5% Donkey serum and 5% BSA in PBS, retinal sections were treated with primary antibodies: custom made rabbit anti-ET_B_ (Antibody Research Corporation, St. Charles, MO) diluted 1∶200 (7.5 µg/ml), and incubated for 1 h at room temperature. Secondary incubation for 1 hr was carried out with a 1∶ 1000 dilution of the appropriate secondary antibody conjugated with Alexa 488 (Molecular Probes, Eugene, OR). Retinal sections in which the primary antibody incubation was excluded served as blanks and were used to assess non-specific staining by the secondary antibody. Fluorescence images were taken in a Zeiss LSM 510 META confocal microscope.

### Receptor Binding Assays

IOP was elevated in the left eye of Brown Norway rats by the method of Morrison et al. (1997), while the corresponding right eye served as contralateral control. After maintaining the rats with elevated IOP for 2 weeks, they were sacrificed and retinas were isolated form left and right eyes. The retinas were homogenized in a solution of 1× TBS (50 mM Tris.HCl, pH 7.4 and 150 mM NaCl) containing protease inhibitors and plasma membrane fractions were isolated by centrifugation at 100, 000 g.

Approximately 3 µg of membrane protein from rat retinas were used for each binding reaction. ^125^I-ET-1 (NEN Life Science Products Inc., Boston, MA, USA) binding was performed in polypropylene tubes in a total assay volume of 90 µl containing 30 µl of ^125^I-ET-1 (ranged from 0.2 to 2 nM), 30 µl of membrane fraction and 30 µl of competition reagent (either cold ET-1 or ET_A_ receptor antagonist) or buffer at 37°C for 1 hr. Binding was terminated by adding 5 ml of cold wash buffer (10 mM Tris.HCl pH 7.4 containing 150 mM NaCl) and binding solution was rapidly vacuum filtered through glass fiber filters (No. 30, Schleicher and Schuell Keene, NH, USA). Filters were washed twice with 5 ml of wash buffer and the bound radioactivity was quantitated in a gamma counter. Non-specific binding was assessed by determining filter-bound radioactivity in the presence of 1 µM unlabeled ET-1 (Bachem, Torrance, CA, USA) after addition of ^125^I-ET-1. The binding of ET_A_ receptor was determined by measuring the decrease in binding in the presence of 200 nM of BQ-610 (ET_A_ receptor antagonist) (Peninsula Lab Inc. Belmont, CA, USA). ET_B_ receptor binding was defined as the total specific ^125^I-ET-1 binding minus the amount of ET_A_ receptor binding. Estimates of maximum number of binding sites (*B*_max_) were obtained using unweighted linear regression analysis of data transformed by the method of Scatchard [36].

### Live-Dead Assay for primary RGC viability

Primary cultures of rat retinal ganglion cells were prepared using a two-step panning procedure [79]. Briefly, post-natal 3–7 day old Sprague Dawley rat pups (30 pups from 3 litters) (Charler River, Wilmington, MA) were euthanized, and the retinas were placed in 4.5 units/mL of papain solution (Worthington, Lakewood, NJ) to dissociate the tissue. This was followed by incubation of cells for 10 min with a rabbit anti-macrophage antibody (Cedarlane, Burlington, Onatario, Canada). After that cell suspensions were incubated in a 150-mm Petri dish coated with a goat anti-rabbit IgG (H+L chain) antibody (Jackson ImmunoResearch, West Grove, PA) for 30 minutes. Cells that did not adhere to the 150-mm dish were then transferred to a 100-mm dish coated with anti-Thy1.1 antibody (from hybridoma T11D7; American Type Culture Collection, Rockville, MD) for 45 minutes. Cells were then trypsinized off (1250 units/mL) (Sigma-Aldrich, St. Louis, MO) the petri dish and plated on coverslips coated with mouse-laminin (Trevigen Inc., Gaithersburg, MD). Then, cells were cultured in a serum free defined media containing BDNF (50 ng/mL) (Peprotech, Rocky Hill, NJ), CNTF (10 ng/mL) (Peprotech, Rocky Hill, NJ), and forskolin (5 ng/mL) (Sigma-Aldrich, St. Louis, MO). Cells were incubated at 37°C in a humidified atmosphere of 10% CO_2_ and 90% air.

After 1 week in culture, primary RGCs were viable and showed good neurite outgrowth. Primary RGCs grown on coverslips were either untreated (control) or were treated with ET-1(10 nM and 100 nM) or ET-3 (100 nM) for 24 hr. The cells were treated with a mixture of green-fluorescent calcein-AM (to indicate intracellular esterase activity of living cells) and red-fluorescent ethidium homodimer-1 (EtHD) (indicative of dead cells) was added to assess the viability of the cells (Live/Dead® Viability/Cytotoxicity Kit, Eugene, OR, USA). Eight images were taken for each treatment condition, in a Zeiss LSM 510 META confocal microscope and the number of viable and dead cells was counted using the image J software. The number of dead cells were expressed as a percentage of total cells in each field of view and mean values of percent of dead cells for each treatment condition was calculated. Statistical analyses were performed by One Way ANOVA to determine if there was a significant increase in cell death in various treatment groups in comparison to the untreated control group.

### Paraphenylenediamine (PPD) staining

IOP elevation was carried out in WT and KO rats [35]. The rats were maintained for 4 weeks after IOP elevation, following which they were sacrificed, eyes enucleated and optic nerves were excised 2 mm posterior to the globe. The optic nerves were fixed with 2% paraformaldehyde, 2.5% glutaraldehyde in 0.1 M sodium cacodylate buffer for 3 hrs at room temperature. After osmification and embedding in epon, optic nerve cross sections were obtained and stained with 1% paraphenylenediamine for 10 min at room temperature by a modification of a published protocol [80]. Images were taken in a Zeiss LSM 510 META confocal microscope. The images were graded in a blinded manner by five individuals giving a score ranging from 0 to 9 by a modification of the method [81]. The grades assigned to each treatment group were compared to determine if there were neuroprotective/neurodegenerative changes between the different groups.

### TUNEL assay for detection of apoptosis

Primary RGCs were isolated from postnatal day 3–7 rat pups using a two step panning procedure (78) and seeded on 12 mm coverslips. The cells were allowed to attach and grow for 1 week till they displayed good neurite outgrowth. The RGCs were either untreated or treated with ET-1 (10 nM), ET-1 (100 nM) or ET-3 (100 nM) for 24 hr. Following treatments, the RGCs on coverslips were fixed with 4% formaldehyde in PBS for 25 min at 4°C. TUNEL assays were carried out using the DeadEnd Fluorometric TUNEL System (Promega, Madison, WI) by the manufacturer's instructions. Briefly, the cells were permeabilized in 0.2% Triton X-100 in PBS for 5 min followed by two washes with PBS. A negative control reaction was carried out by incubating one coverslip of RGCs with fluorescein-12-dUTP in the absence of TdT enzyme. Another coverslip was treated with DNase I enzyme to introduce DNA cleavage prior to the incubation with the reaction mix. The labeling reaction was carried out by incubating with a mixture of fluorescein-12-dUTP and terminal deoxynucleotidyl transferase (TdT) at for 60 min at 37°C. Following the incubation, the cells were washed with PBS and incubated with 4′ 6 Diamidino-phenylindole dichloride (DAPI) to stain nuclei. The TUNEL positive cells were detected by incorporation of fluorescein and fluorescent images were taken in an EVoS microscope.

## Supporting Information

Figure S1 **Experimental controls for TUNEL assay of primary RGCs.** Primary RGCs were either untreated (top horizontal panel) or treated with DNAse I as a positive control (middle horizontal panel). TUNEL assay was carried out using a combination of terminal deoxynucleotidyl transferase (TdT) and fluorescein-12-dUTP. Another set of RGCs were subjected to the negative control reaction by treatment with fluorescein-12-dUTP alone, with the exclusion of TdT (lower horizontal panel). The left vertical panel (TUNEL) indicates fluorescent images from cells incorporating fluorescein-12-dUTP indicative of apoptosis. The right vertical panel (DAPI) shows stained nuclei using DAPI. Scale bar indicates 400 µm.
(TIF)
