# Supplementary figures and images for: Endothelin B Receptors Contribute to Retinal Ganglion Cell Loss in a Rat Model of Glaucoma
Source: PLoS One. 2012 Aug 20;7(8):e43199. doi: 10.1371/journal.pone.0043199 (PMC3423444; doi:10.1371/journal.pone.0043199)

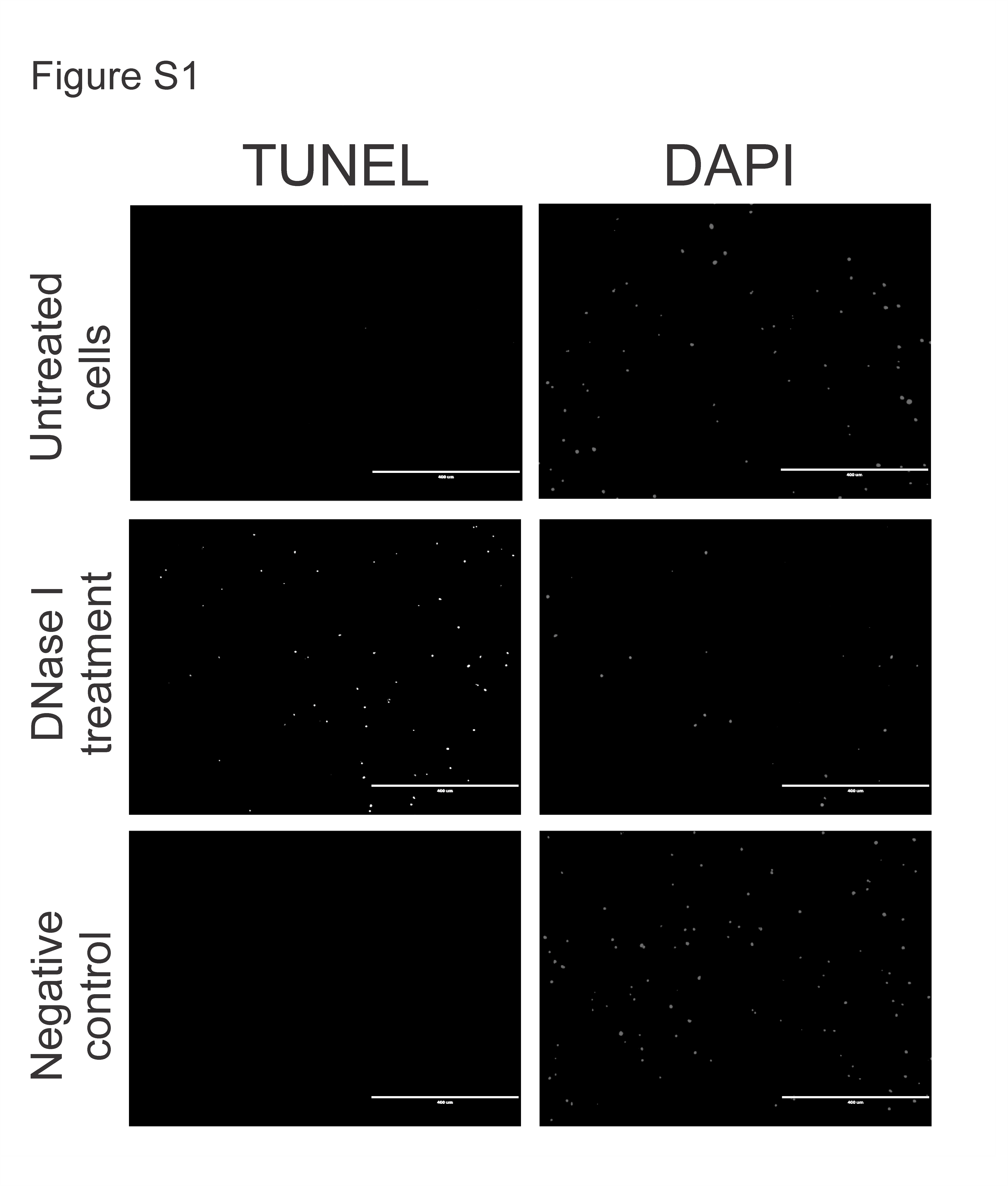

Supplement: Figure S1 — Experimental controls for TUNEL assay of primary RGCs. Primary RGCs were either untreated (top horizontal panel) or treated with DNAse I as a positive control (middle horizontal panel). TUNEL assay was carried out using a combination of terminal deoxynucleotidyl transferase (TdT) and fluorescein-12-dUTP. Another set of RGCs were subjected to the negative control reaction by treatment with fluorescein-12-dUTP alone, with the exclusion of TdT (lower horizontal panel). The left vertical panel (TUNEL) indicates fluorescent images from cells incorporating fluorescein-12-dUTP indicative of apoptosis. The right vertical panel (DAPI) shows stained nuclei using DAPI. Scale bar indicates 400 µm. (TIF) [file pone.0043199.s001.tif]
